# Time-Dependent Changes in Salivary Antioxidants After 5-ALA Photodynamic Therapy vs. Clobetasol in Oral Lichen Planus: A Randomized Clinical Trial

**DOI:** 10.3390/ijms262211232

**Published:** 2025-11-20

**Authors:** Patryk Wiśniewski, Magdalena Sulewska, Jagoda Tomaszuk, Anna Zalewska, Sara Zięba, Aleksandra Pietruska, Emilia Szymańska, Katarzyna Winnicka, Mateusz Maciejczyk, Małgorzata Żendzian-Piotrowska, Małgorzata Pietruska

**Affiliations:** 1 Department of Periodontal and Oral Mucosa Diseases, Medical University of Bialystok, ul. Waszyngtona 13, 15-269 Białystok, Poland; magdalena.sulewska@umb.edu.pl (M.S.); jagoda.tomaszuk@umb.edu.pl (J.T.);; 2 Department of Restorative Dentistry, Medical University of Bialystok, ul. Marii Skłodowskiej Curie 24a, 15-089 Białystok, Poland; anna.zalewska1@umb.edu.pl (A.Z.); sara.zieba@umb.edu.pl (S.Z.); 3 Student’s Research Group, Department of Periodontal and Oral Mucosa Diseases, Medical University of Bialystok, ul. Waszyngtona 13, 15-269 Białystok, Poland; perio@umb.edu.pl; 4 Department of Pharmaceutical Technology, Medical University of Bialystok, Mickiewicza 2c, 15-222 Białystok, Poland; emilia.szymanska@umb.edu.pl (E.S.); katarzyna.winnicka@umb.edu.pl (K.W.); 5 Department of Hygiene, Epidemiology and Ergonomics, Medical University of Bialystok, Mickiewicza 2c, 15-222 Białystok, Poland; mat.maciejczyk@gmail.com (M.M.); mzpiotrowska@gmail.com (M.Ż.-P.)

**Keywords:** oral lichen planus, photodynamic therapy, clobetasol propionate, molecular biomarkers, glutathione, oxidative stress

## Abstract

In this randomized clinical trial, we compared the effects of 5-aminolevulinic acid photodynamic therapy (ALA-PDT) and topical clobetasol on the salivary antioxidant profile in patients with oral lichen planus (OLP) and explored their relationships with clinical outcomes. Ninety adults with OLP were randomly allocated to ALA-PDT (five weekly sessions) or clobetasol (twice daily for 14 days). Unstimulated whole saliva was collected at baseline (T0), immediately after treatment (T1), and at 3 (T3) and 6 months (T6). The activities of catalase (CAT), superoxide dismutase (SOD), peroxidase (Px) and reduced glutathione (GSH) were determined, and nonparametric statistics were applied, including Friedman tests with Dunn’s post hoc comparisons and Spearman’s rank correlations. Both therapies induced an early decline in CAT, Px and GSH at T1, followed by partial recovery at later time points. SOD activity changed significantly over time in the clobetasol group, but not in the PDT arm. At T6, Px and GSH remained below baseline in both groups despite improvement from the immediate post-treatment nadir. No significant between-group differences were observed at individual time points, although GSH at T6 showed a non-significant trend favoring PDT. Exploratory analyses revealed modest, treatment-dependent associations between salivary antioxidant activity and lesion size, as well as between the former and pain intensity. Overall, ALA-PDT and topical clobetasol both modulated the salivary redox profile, primarily through short-term depletion of enzymatic and non-enzymatic antioxidants with incomplete recovery over 6 months, and no clear redox superiority of one modality over the other was demonstrated. These findings are hypothesis-generating and underscore the need for larger, longer-term studies with broader redox panels and more advanced between-group analyses.

## 1. Introduction

**Figure 1:**
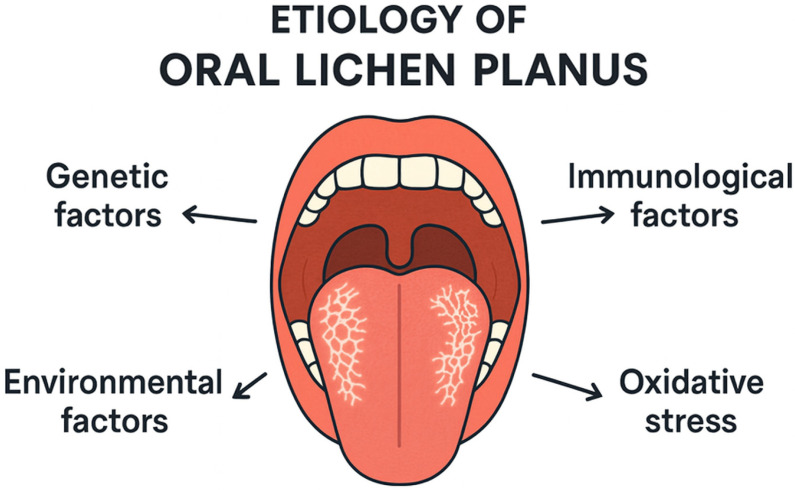
Etiological factors of OLP.

Oral lichen planus (OLP) is a chronic, autoimmune disease of the oral mucosa classified among the oral potentially malignant disorders (OPMDs). It is estimated that OLP affects approximately 0.89–1.01% of the general population, with a clear predominance among women over 40 years of age [1,2]. Clinically, the lesions present in various forms, ranging from reticular and papular, through erythematous, to erosive, which is associated with the most severe pain symptoms and an increased risk of malignant transformation into oral squamous cell carcinoma (OSCC) [3,4]. The etiopathogenesis of OLP has not yet been fully elucidated. A key role is played by an abnormal immune response leading to the activation of cytotoxic CD8+ T lymphocytes and the induction of keratinocyte apoptosis [5,6]. Genetic factors are also considered important, including variants of major histocompatibility complex (MHC) genes, which predispose individuals to aberrant antigen presentation and an intensified inflammatory response [7,8]. The development of the disease is additionally influenced by numerous environmental factors, such as chronic infections (particularly hepatitis C virus), tobacco use, alcohol consumption, psychological stress, and exposure to certain medications (e.g., beta-blockers, NSAIDs), as well as contact with dental amalgam [9,10,11,12,13,14,15] (Figure 1).

An increasing body of evidence indicates that oxidative stress (OS) plays a significant role in the pathogenesis of OLP [16,17,18]. Excessive production of reactive oxygen species (ROS) by inflammatory cells leads to damage of keratinocyte lipid membranes, proteins, and nucleic acids, thereby enhancing apoptosis and perpetuating chronic inflammation [16,17,19]. Elevated levels of lipid peroxidation products and decreased activity of antioxidant enzymes have also been confirmed in patients with OLP [20,21].

Saliva is an easily accessible and non-invasive material for evaluating oxidative stress biomarkers [22,23]. In patients with OLP, increased concentrations of lipid and DNA oxidation products such as malondialdehyde (MDA) and 8-hydroxy-2′-deoxyguanosine (8-OHDG) have been reported, along with decreased activity of key antioxidant enzymes, including catalase (CAT), superoxide dismutase (SOD), peroxidase (Px), and reduced glutathione (GSH) [23,24,25,26,27,28,29,30,31,32,33]. Moreover, other studies have also assessed global oxidative stress indices, showing that total oxidant status (TOS) is significantly elevated in OLP, while total antioxidant capacity (TAC) in the blood of OLP patients is markedly reduced compared to that in healthy controls [18,34,35]. Disturbances in the redox balance of saliva may correlate with disease activity and the potential risk of malignant transformation.

Topical application of glucocorticosteroids (GKSs) remains the gold standard for the symptomatic management of OLP, as these agents effectively reduce pain intensity and lesion size [36,37,38]. However, long-term GKS therapy is associated with numerous adverse effects, including oral fungal infections, mucosal atrophy, delayed healing, and impaired salivary secretion [39,40,41]. In recent years, photodynamic therapy (PDT) has gained increasing attention as a minimally invasive treatment alternative with a favorable safety profile. PDT involves topical application of a photosensitizer, most commonly 5-aminolevulinic acid (5-ALA), followed by activation with light of an appropriate wavelength, resulting in the generation of reactive oxygen species and selective destruction of pathologically altered cells [42,43,44,45,46,47,48,49,50,51,52,53,54,55,56]. In a recent randomized clinical trial from our research group, PDT achieved very good clinical outcomes and was at least as effective as topical glucocorticosteroids, with sustained reductions in lesion size and pain intensity [43]. Importantly, PDT is associated with a low incidence and typically mild intensity of adverse effects and can be used in patients with contraindications to topical or systemic corticosteroids. PDT has therefore emerged as a promising therapeutic option for OLP, combining high efficacy with good tolerability and an improved safety profile [43,44,45,46,47,48].

Evaluating the impact of therapeutic interventions on oxidative stress parameters in saliva is of substantial importance in the search for strategies that can extend remission periods and reduce the risk of relapse and progression to potentially malignant lesions. Our previous work in this cohort demonstrated significant alterations in global redox indices after treatment, highlighting a pronounced redox imbalance in OLP and suggesting that therapy-induced modulation of oxidative stress may contribute to clinical improvement [57]. However, it remains unclear which specific antioxidant systems respond most sensitively to therapy and whether changes in salivary antioxidant activity are associated with the magnitude of clinical benefit.

Therefore, the aim of the present study was twofold: (i) to compare the effects of photodynamic therapy and topical corticosteroid therapy on the activity of selected salivary antioxidants (SOD, CAT, Px, GSH) in patients with OLP in a randomized clinical trial setting, and (ii) to assess the correlations between salivary antioxidant activity and changes in lesion size and pain intensity (VAS) according to the applied treatment modality.

## 2. Results

A total of 90 patients with OLP were included in the study, comprising 72 women and 18 men (Table 1). The participants were randomized into the photodynamic therapy (PDT) group and the topical corticosteroid therapy (GKS) group. Changes in the activity of antioxidant enzymes and the concentration of reduced glutathione in unstimulated saliva were assessed at four time points: before treatment (PDT0, GKS0), immediately after completion of treatment (PDT1, GKS1), three months after treatment (PDT3, GKS3), and six months after treatment (PDT6, GKS6).

### 2.1. Patients Treated with PDT

**Figure 2:**
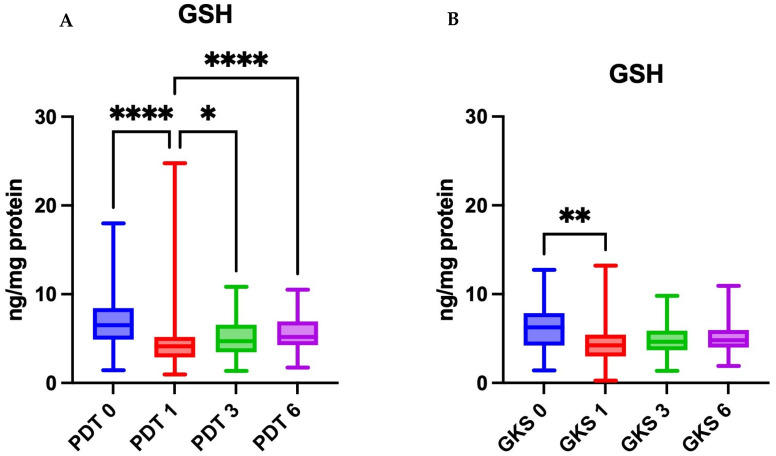
The concentration of reduced glutathione over time in the groups treated with photodynamic therapy (**A**) and corticosteroid therapy (**B**). GSH—reduced glutathione. PDT0, GKS0—before treatment; PDT1, GKS1—immediately after treatment; PDT3, GKS3—after 3 months; PDT6, GKS6—after 6 months. * *p* < 0.05, ** *p* < 0.01, **** *p* < 0.0001. [GSH] = ng/mg protein. Data are presented as medians with interquartile ranges.

**Figure 3:**
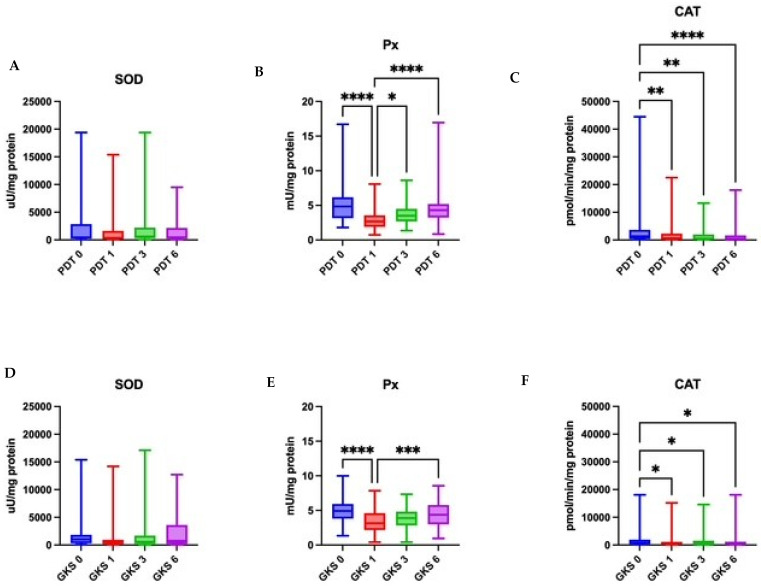
Catalase, peroxidase, and superoxide dismutase activity over time in the groups treated with photodynamic therapy (**A**–**C**) and corticosteroid therapy (**D**–**F**). SOD—superoxide dismutase; CAT—catalase; Px—peroxidase. PDT0, GKS0—before treatment; PDT1, GKS1—immediately after treatment; PDT3, GKS3—after 3 months; PDT6, GKS6—after 6 months. * *p* < 0.05, ** *p* < 0.01, *** *p* < 0.001, **** *p* < 0.0001. [SOD] = µU/mg protein, [CAT] = pmol/min/mg protein, [Px] = mU/mg protein. Data are presented as medians with interquartile ranges.

The median SOD activity before treatment was 455 µU/mg protein. Subsequent measurements showed fluctuations; however, the changes were not statistically significant (Friedman *p* = 0.2723) (Table 2 and Table S1). The median CAT activity prior to therapy was 13,335 pmol/min/mg protein and significantly decreased after treatment to 664.8 pmol/min/mg (post hoc T1 vs. T0 *p* < 0.01), remaining at a lower level at 3 and 6 months of follow-up (Table 3 and Table S2). The median Px activity before treatment was 4.836 mU/mg protein and significantly decreased to 2.679 mU/mg after therapy (*p* < 0.0001). In subsequent months, Px activity increased but still differed significantly from baseline values (*p* < 0.05 and *p* < 0.0001) (Table 4 and Table S3). The median GSH concentration before treatment was 6.514 ng/mg protein. After therapy, it decreased significantly to 4.129 ng/mg (*p* < 0.0001) and partially increased in later assessments, while maintaining statistically significant differences compared to baseline (*p* < 0.05 and *p* < 0.0001) (Table 5 and Table S4; Figure 2 and Figure 3).

### 2.2. Patients Treated with Topical Corticosteroids

In the GKS arm, the median SOD activity before treatment was 1022 µU/mg protein. After therapy, values declined to 366.4 at T1 and then increased to 573.2 at T3 and 764.9 at T6; the overall time effect was significant (Friedman *p* = 0.0338) (Table 2 and Table S1). The median CAT activity prior to treatment was 739.3 pmol/min/mg protein and decreased after therapy to 483.0 at T1, remaining lower at T3 (324.3) and T6 (375.6); the time effect was significant (Friedman *p* = 0.0044) (Table 3 and Table S2). The median peroxidase activity before treatment was 4.917 mU/mg protein and decreased to 3.155 at T1, with partial recovery at T3 (3.885) and T6 (4.377); the overall time effect was significant (Friedman *p* < 0.0001) (Table 4 and Table S3). The median GSH concentration before treatment was 6.263 ng/mg protein and declined to 4.232 at T1, with partial increases at T3 (4.638) and T6 (4.809); the time effect was significant (Friedman *p* = 0.004) (Table 5 and Table S4; Figure 2 and Figure 3).

### 2.3. Comparison Between PDT and Corticosteroid Groups

The activities of SOD, CAT, and Px did not differ significantly between the groups at any time point of the study (*p* > 0.05). The GSH concentration did not differ significantly between the groups (*p* > 0.05). At the six-month follow-up, a non-significant trend toward higher salivary GSH levels was observed in patients treated with photodynamic therapy compared to those receiving corticosteroid therapy (*p* = 0.0893). (Table 6)

### 2.4. *Correlations Between Salivary Enzyme Activity and Clinical Outcomes by Treatment Modality*

Median lesion area and VAS scores were comparable between the PDT and GKS groups at baseline (T0). Both treatment modalities led to a reduction in lesion size and pain immediately after therapy (T1), with the effect partially maintained at the 6-month follow-up (T6). These data provide the clinical context for the correlation analyses between salivary redox parameters and treatment outcomes and have been reported in detail in a previously published study from our research group (Table 7 and Table 8) [43].

In the PDT group, no significant associations were observed at baseline between lesion size or VAS and the activities of SOD, Px, CAT or GSH. Immediately after PDT (T1), higher CAT activity correlated moderately with smaller lesion area (r = −0.282, *p* = 0.047) and lower pain intensity (r = −0.311, *p* = 0.028). In addition, higher GSH activity was associated with lower VAS scores (r = −0.288, *p* = 0.043). At the 6-month follow-up (T6), the correlations between salivary enzyme activity and clinical parameters were no longer significant (Table 9).

In the GKS group, baseline VAS scores showed a moderate positive correlation with Px (r = 0.341, *p* = 0.031) and GSH activity (r = 0.338, *p* = 0.033), whereas correlations with lesion size were weak and non-significant. After glucocorticosteroid therapy (T1) and at T6, no statistically significant correlations were found between lesion size or VAS and the activities of SOD, Px, CAT or GSH (Table 10).

## 3. Discussion

Oral lichen planus is a chronic condition that remains difficult to manage. Current therapies primarily alleviate symptoms and dampen inflammation rather than directly modifying the underlying pathogenic mechanisms. Consequently, there is continued interest in adjunctive or alternative strategies that might not only control symptoms but also favorably influence biological processes implicated in disease persistence.

Glucocorticosteroids are regarded as the clinical standard for OLP because they reduce pain and decrease lesion size. However, adverse effects such as mucosal thinning, xerostomia, and secondary fungal infections are not uncommon [39]. Photodynamic therapy has therefore gained attention as a minimally invasive option that is generally well tolerated and may support longer remissions [58,59].

Since chronic oxidative stress is thought to contribute to keratinocyte apoptosis and sustained mucosal inflammation in OLP, salivary redox readouts can offer insight into treatment-related biological modulation. Prior reports indicate that patients with OLP differ from healthy controls in several salivary redox parameters [30,31,32].

In this randomized comparison, both therapeutic approaches produced a broadly similar temporal pattern across multiple markers: an immediate post-treatment decline followed by partial recovery during follow-up.

After corticosteroid therapy, CAT decreased and remained lower over time; SOD exhibited a statistically significant but modest time effect; Px decreased immediately post-treatment with partial rebound. GSH decreased after treatment and stayed below baseline at subsequent assessments.

After PDT, CAT also fell and stayed reduced. SOD did not change significantly; Px declined then increased over time; and GSH dropped immediately after therapy and showed a tendency to increase by six months.

When the two strategies were compared with respect to redox modulation, most parameters (CAT, SOD, Px) did not differ significantly between groups at long-term follow-up. At six months, GSH in the PDT arm showed a non-significant trend toward higher values compared with corticosteroids (*p* = 0.0893).

This trend should be interpreted cautiously: despite the rise relative to the immediate post-treatment nadir, GSH values at six months remained below baseline, indicating only a partial restoration of the glutathione pool. Biologically, a late-phase increase in reduced GSH could reflect either enhanced regeneration of GSSG to GSH via glutathione reductase or increased de novo synthesis through the γ-glutamylcysteine pathway [60,61,62]. Because neither glutathione reductase activity nor total glutathione (GSH + GSSG) was measured, the mechanism underlying the observed pattern cannot be specified here and remains a limitation of the study.

The therapeutic mechanisms are consistent with the observed biochemical trajectories. Corticosteroids reduce pro-inflammatory cytokine expression and T-cell activity [37,38,63]; attenuating inflammation may secondarily lower reactive oxygen species (ROS) production and thereby alter antioxidant enzyme activity. PDT generates ROS within diseased tissue, induces apoptosis of pathological cells, reduces lesion burden [50,51], and can have immunomodulatory effects—such as lowering the abundance of activated CD137+ T lymphocytes—which may help limit chronic inflammation and contribute to re-balancing the redox milieu [64].

Beyond group-level changes, the correlation analyses between salivary antioxidant activity and clinical outcomes provide additional mechanistic insight. In the PDT group, higher CAT activity at the end of therapy (T1) correlated moderately with both smaller lesion area and lower pain intensity, while increased GSH activity was also associated with reduced VAS scores. These findings suggest that patients who are able to mount a stronger antioxidant response, especially in terms of CAT and GSH activity, may experience greater clinical benefit from ALA-mediated PDT. Given that PDT induces a controlled burst of ROS, an efficient salivary antioxidant defense could help to contain oxidative damage to diseased tissue, limit collateral injury, and thereby favor better symptomatic and morphological improvement. In this context, CAT and GSH behave as candidate indicators of individual responsiveness to PDT rather than simple markers of disease presence.

In contrast, the pattern observed in the corticosteroid arm was different. Before GKS therapy, higher Px and GSH activities correlated positively with pain intensity, whereas associations with lesion size were weak and non-significant. This suggests that elevated salivary antioxidant activity at baseline reflects a higher inflammatory and oxidative burden and thus greater subjective disease severity. After treatment, these correlations disappeared and no consistent associations between enzyme activity and lesion area or VAS were detected at T1 or T6, indicating that, in the context of GKS, clinical improvement is largely decoupled from the salivary antioxidant profile. In other words, antioxidants act primarily as markers of baseline disease load rather than predictors or mediators of corticosteroid response.

Taken together, these exploratory findings suggest that the pattern of associations between salivary redox biomarkers and clinical outcomes may differ depending on the therapeutic modality. In ALA-PDT, higher CAT and GSH activity at the end of therapy showed modest correlations with smaller lesion size and lower pain intensity, which may indicate that inter-individual variability in antioxidant responses is loosely related to the magnitude of clinical benefit. In the GKS group, baseline Px and GSH activity correlated positively with pain, which is more compatible with a role as markers of initial disease burden rather than determinants of treatment response. Given the small effect sizes and multiple comparisons, these results should be regarded as hypothesis-generating rather than definitive. They nonetheless raise the possibility that salivary antioxidant profiling could, after independent confirmation in larger cohorts, contribute to a more nuanced understanding of how different treatment modalities interact with redox homeostasis in OLP.

Nevertheless, the magnitude of change in individual markers was modest and variable, and the partial rebounds observed by three to six months did not translate into clear between-group differences. These findings argue for complementing enzyme-specific measurements with broader indices that capture global redox status in saliva, such as total antioxidant capacity (TAC), total oxidant status (TOS), and the oxidative stress index (OSI) [65,66,67]. They also highlight the value of quantifying total glutathione and related enzyme activities to contextualize changes in reduced GSH. Finally, longer follow-up and denser early sampling could help characterize short-term biochemical dynamics immediately after treatment and clarify their relationship to clinical outcomes.

This study has limitations. The observation window was relatively short, no untreated OLP control group was included for ethical reasons, and differences in methodology across published datasets preclude direct comparisons with healthy controls. The choice of time points (T0, T1, T3, T6) was designed to capture immediate and medium term effects while maintaining feasibility and adherence- more frequent sampling in the early phase was not practical due to financial limitations. Moreover, correlation analyses between antioxidant activity and clinical parameters were performed only for T0, T1 and T6, so the lack of a mid term correlation assessment at T3 may have obscured transient relationships between salivary redox markers and clinical outcomes. In addition, meaningful comparison with previously published work is challenging because, to the best of our knowledge, no studies with a similar design combining a randomized comparison of PDT versus corticosteroids with longitudinal assessment of salivary redox biomarkers and clinical correlations are currently available. The present project should therefore be regarded as a pilot study, and the observed patterns need to be confirmed and refined in larger, independently recruited cohorts.

On this basis, future research should be planned to specifically overcome these limitations. Multicenter randomized controlled trials with larger and more heterogeneous OLP populations and longer observation periods (at least 12 to 24 months) are needed to verify the durability of the observed salivary redox changes and their relationship with remission and relapse. Such trials should include parallel arms with ALA PDT and topical corticosteroids, with or without combination regimens, and, where ethically feasible, an external comparator group of healthy controls. Saliva sampling should be more intensive in the early post treatment phase (for example at 24 to 72 h and at 1 month) and should include a broader panel of biomarkers, encompassing also TAC, TOS, OSI, total glutathione and selected markers of oxidative damage to lipids, proteins and DNA. Finally, future studies should prospectively integrate these biochemical measures with lesion area, pain scores, validated oral health related quality of life outcomes and relapse related endpoints in longitudinal analyses, in order to formally test whether specific redox profiles can serve as prognostic or monitoring tools for different therapeutic modalities in OLP.

## 4. Materials and Methods

### 4.1. Study Participants

The study was conducted as a single-center, prospective, randomized clinical trial at the Department of Periodontal and Oral Mucosa Diseases, Medical University of Bialystok, between September 2021 and January 2023. The study protocol received approval from the Bioethics Committee of the Medical University of Bialystok (decision no.: APK.002.372.2021). All participants were thoroughly informed about the objectives and procedures of the study and provided written informed consent. The study was designed, conducted, and reported in accordance with the Consolidated Standards of Reporting Trials (CONSORT 2010).

A total of 100 individuals with clinically and histopathologically confirmed OLP were enrolled. After applying exclusion criteria, data from 90 patients (72 women and 18 men) aged 29 to 88 years (mean age: 60 ± 11.7 years) were included in the statistical analysis. In total, 161 lesions were identified. Inclusion criteria comprised age over 18 years and a diagnosis of OLP confirmed by histopathological examination. Exclusion criteria included pregnancy, breastfeeding, severe systemic diseases (including oncological, dermatological, and hepatic disorders), known hypersensitivity to light or the photosensitizer, prior OLP treatment within the last 6 months, use of immunosuppressive or immunomodulatory drugs, mental illnesses, and the presence of other oral mucosal diseases.

### 4.2. Study Groups

**Figure 4:**
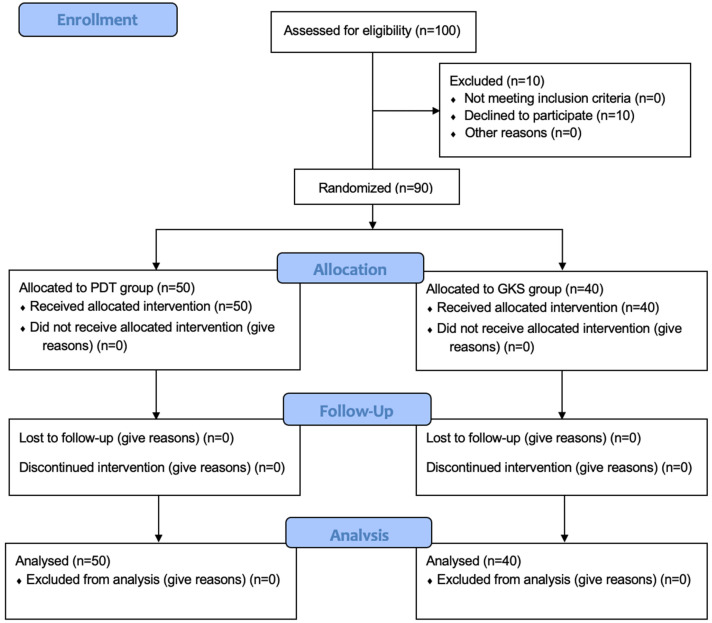
Patient’s flow chart.

Participants were assigned to one of the two treatment arms through simple randomization, based on a pre-prepared allocation list generated in Microsoft Excel for Microsoft 365 (Figure 4). The randomization process was conducted by a single independent investigator. The trial used a single-blind design, in which the clinical examiner remained blinded. This examiner did not have access to information about the type of treatment provided and was instructed not to ask participants about their therapy. Likewise, patients were advised not to reveal details of the procedure they underwent. Full blinding of participants was not possible because the two interventions differed in nature and application.

The PDT group received novel mucoadhesive composition in the form of emulgel containing 5% (*w*/*w*) 5-aminolevulinic acid (5-ALA) (patent P.443813) according to the previously described protocol (ALA-PDT) [68,69]. In brief, after drying the oral mucosa, the 5-ALA preparation was applied to the lesion and the adjacent mucosa in a layer approximately 2 mm thick, twice: 40 and 20 min prior to planned illumination. The treated area was covered with an occlusive dressing made of gauze and secured with sterile compresses to limit the access of saliva. Illumination was performed using an LED light source (FotoSan^®^ 630, CMS Dental A/S, Roslev, Denmark) emitting light at a wavelength of 630 nm, with a power output of 300 mW and an energy density of 108 J/cm^2^. The beam was delivered in non-contact mode at approximately 2 mm from the lesion, applied in a single continuous stage without interruptions for 6 min per square centimeter of the lesion. The complete treatment protocol included five individual sessions, each performed once a week over a period of five consecutive weeks.

The second group was treated with a topical corticosteroid—clobetasol propionate (Clobederm 0.5 mg/g), applied twice daily for 14 days.

### 4.3. Clinical Data

Clinical evaluation was performed at three time points: before the initiation of therapy (T0), immediately after completion of the treatment protocol (T1), and at the 6-month follow-up visit (T6). At each visit, macroscopic assessment of the lesions was accompanied by standardized photographic documentation.

Lesion size was determined using a periodontal probe (PCPUNC 15; Hu-Friedy). For each lesion, the greatest length and width were recorded as the longest distances between the most peripheral points of the lesion and the border of clinically healthy mucosa. These two dimensions were then used to calculate the lesion area, which was expressed in cm^2^.

All clinical measurements were obtained by a single examiner who was blinded to group allocation. Prior to the study, the examiner underwent calibration on a separate group of 10 patients not included in the trial. For calibration purposes, duplicate measurements were taken 24 h apart, and the allowable discrepancy between the two readings was set at ≤0.5 cm.

In addition, patients completed a questionnaire addressing their subjective symptoms related to the oral lesions. The intensity of pain, burning, and itching was rated using a visual analog scale (VAS). For descriptive purposes, the VAS scores were categorized as follows: 0—no symptoms; 1–3—mild symptoms; 4–6—moderate/marked symptoms; 7–9—very severe symptoms; and 10—the worst pain imaginable [70].

### 4.4. Saliva Collection

Unstimulated saliva samples were collected at four time points: directly before the initiation of therapy (T0), at the end of each treatment protocol (T1–corresponding to 5 weeks after PDT initiation and 2 weeks after corticosteroid initiation), and during follow-up at 3 months (T3) and 6 months (T6) after the completion of each treatment. Strict pre-collection instructions were provided: participants refrained from eating and drinking (except water) for at least two hours prior to sampling, did not use oral hygiene products, and avoided taking medications for at least eight hours. Sampling was performed in the morning hours (between 8:00 and 10:00 a.m.) in a separate room, with the patient seated and the head tilted downward, after a 5 min adaptation period. The oral cavity was rinsed with distilled water, and the first minute of salivation was discarded. Subsequent portions were collected by expectoration over 15 min until a volume of 5 mL was obtained. Samples were placed in Falcon tubes, kept on ice, centrifuged (4 °C, 3000× *g*, 20 min), and then frozen at −80 °C until analysis.

### 4.5. Biomarker Assays

The saliva samples were analyzed for the activity of selected antioxidant enzymes: catalase, superoxide dismutase, salivary peroxidase and the concentration of reduced glutathione. All measurements were performed using colorimetric methods.

Catalase activity was determined according to Aebi’s method, which measures the rate of hydrogen peroxide decomposition at a wavelength of 240 nm [71]. Superoxide dismutase activity was assessed according to Misra’s method, based on the inhibition of adrenaline autoxidation at 320 nm [72]. Salivary peroxidase activity was determined by the Mansson-Rahemtulla method through DTNB reduction in the presence of potassium thiocyanate and hydrogen peroxide, with absorbance measured at 412 nm [73]. The concentration of reduced glutathione was measured by Ellman’s method using DTNB reagent, with absorbance recorded at 412 nm [74].

All results were normalized to total protein content and expressed as enzymatic activity units per milligram of protein. Total protein content was determined using the bicinchoninic acid assay (Thermo Scientific PIERCE BCA Protein Assay kit (Rockford, IL, USA).

### 4.6. Statistical Analysis

The required sample size was calculated using G*Power 3.1 software (Universität Düsseldorf, Düsseldorf, Germany) based on pilot data from a preliminary study. Assuming a medium effect size (f = 0.25), a significance level of α = 0.05, and a statistical power of 0.80 for repeated measures ANOVA with a within–between interaction design (two groups × four time points), the minimum required sample size was 86 participants. Anticipating a dropout rate of approximately 10%, the target enrollment was increased to 100 individuals. Statistical analysis was performed using GraphPad Prism version 10.5 (GraphPad Software, La Jolla, CA, USA). The Shapiro–Wilk test was used to assess the normality of distribution, and Levene’s test was used to assess the homogeneity of variances. Analyses showed that the data did not meet the assumptions of normal distribution; therefore, nonparametric tests were applied. Due to the very large differences between minimum and maximum values, interpretation of mean values was limited. Therefore, statistical analyses were performed on medians, which are less sensitive to distortions caused by extreme values. The Friedman repeated measures analysis of variance by ranks was used, with Dunn’s test employed as a post hoc procedure. Correlations between lesion size, pain intensity (VAS), and salivary antioxidant activity were assessed using Spearman’s rank correlation coefficient. Corrections for multiple comparisons were applied. The level of statistical significance was set at *p* < 0.05.

## 5. Conclusions

In this randomized clinical trial, both ALA-mediated photodynamic therapy and topical clobetasol led to an early post-treatment decrease in salivary antioxidant activity, with only partial recovery over a 6-month follow-up. SOD changed significantly over time only in the corticosteroid group, whereas neither therapy produced sustained improvements in CAT or Px. GSH showed a non-significant tendency to be higher in the PDT arm at 6 months, but remained below baseline in both treatment groups.

Exploratory correlation analyses suggested modest, treatment-dependent associations between salivary antioxidant activity and clinical outcomes: higher CAT and GSH activity after PDT were weakly related to smaller lesion size and lower pain, while baseline Px and GSH in the clobetasol group were positively associated with pain intensity. These findings should be interpreted with caution and regarded as hypothesis-generating. No clear long-term redox superiority of one modality over the other was demonstrated. Future studies with larger cohorts, extended follow-up and broader redox panels are needed to confirm these patterns, clarify the clinical relevance of salivary redox markers, and determine whether they can contribute to more individualized therapeutic strategies in OLP.

## 6. Patents

Szymańska et al. (2023) [68,69], A pharmaceutical composition with mucoadhesive properties and its use. P.443813 (PL); PCT/IB2024/051420–PCT application number.

## Figures and Tables

**Table 1:** Descriptive statistics for age and gender.

Variable	N (%)
Gender	
Male	18 (20%)
Female	72 (80%)
Age	
Average age (M ± SD)	60 ± 11.7 years

**Table 2:** Descriptive statistics of superoxide dismutase (SOD) activity in the corticosteroid-treated group (GKS) and photodynamic therapy group (PDT) at T0, T1, T3, and T6.

	GKS					PDT				
	Mean ± SD	95% CI	Median	Min–Max	Friedman p	Mean ± SD	95% CI	Median	Min–Max	Friedman p
T0	2065 ± 3239	1029–3101	1022	78.66–15,389	0.0338	2081 ± 3443	1102–3059	455	0–19,392	0.2723
T1	1385 ± 3021	419.2–2352	366.4	87.57–14,207		1747 ± 3312	806.1–2689	331.5	34.21–15,389	
T3	1786 ± 3198	763.2–2809	573.2	11.4–17,109		2681 ± 4439	1419–3943	608.6	26.35–19,392	
T6	2731 ± 3844	1501–3960	764.9	16.96–12,721		1564 ± 2412	878.2–2249	478.1	30.05–9521	

**Table 3:** Descriptive statistics of catalase (CAT) activity in the corticosteroid-treated group (GKS) and photodynamic therapy group (PDT) at T0, T1, T3, and T6.

	GKS					PDT				
	Mean ± SD	95% CI	Median	Min–Max	Friedman p	Mean ± SD	95% CI	Median	Min–Max	Friedman p
T0	1971 ± 3886	728.5–3214	739.3	16.85–18,114	0.0044	4096 ± 7536	1954–6237	1335	55.71–44,498	<0.0001
T1	1938 ± 3850	706.9–3169	483	33.63–15,173		1856 ± 3664	814.9–2898	664.8	38.82–22,508	
T3	2183 ± 4174	848–3518	324.3	43.28–14,590		1851 ± 3130	961.8–2741	461.6	18.76–13,323	
T6	2246 ± 4647	759.8–3732	375.6	8.02–18,114		1919 ± 3808	837.2–3001	327.2	44.59–18,015	

**Table 4:** Descriptive statistics of salivary peroxidase (Px) activity in the corticosteroid-treated group (GKS) and photodynamic therapy group (PDT) at T0, T1, T3, and T6.

	GKS					PDT				
	Mean ± SD	95% CI	Median	Min–Max	Friedman p	Mean ± SD	95% CI	Median	Min–Max	Friedman p
T0	5.111 ± 2.034	4.46–5.761	4.917	1.334–9.989	<0.0001	5.135 ± 2.587	4.4–5.87	4.836	1.815–16.71	<0.0001
T1	3.453 ± 1.728	2.901–4.006	3.155	0.4126–7.849		3.027 ± 1.608	2.57–3.484	2.679	0.7496–8.068	
T3	3.965 ± 1.582	3.459–4.471	3.885	0.4027–7.344		3.68 ± 1.463	3.264–4.096	3.502	1.355–8.63	
T6	4.354 ± 1.689	3.814–4.894	4.377	0.9547–8.553		4.745 ± 2.72	3.972–5.518	4.313	0.8585–16.96	

**Table 5:** Descriptive statistics of reduced glutathione (GSH) concentration in the corticosteroid-treated group (GKS) and photodynamic therapy group (PDT) at T0, T1, T3, and T6.

	GKS					PDT				
	Mean ± SD	95% CI	Median	Min–Max	Friedman p	Mean ± SD	95% CI	Median	Min–Max	Friedman p
T0	6.09 ± 2.796	5.196–6.984	6.263	1.424–12.73	0.004	6.691 ± 3.177	5.788–7.593	6.514	1.429–17.98	<0.0001
T1	4.476 ± 2.289	3.744–5.208	4.232	0.2613–13.21		4.528 ± 3.421	3.556–5.5	4.129	0.9584–24.75	
T3	4.806 ± 1.833	4.22–5.392	4.638	1.357–9.811		5.108 ± 2.242	4.471–5.745	4.705	1.353–10.83	
T6	4.997 ± 1.754	4.436–5.558	4.809	1.901–10.94		5.731 ± 2.023	5.156–6.306	5.179	1.749–10.51	

**Table 6:** Comparison of median activities of antioxidant enzymes and GSH concentration in unstimulated saliva of patients treated with photodynamic therapy and topical corticosteroids at each time point.

Parameter	Time Point	PDT Median	GKS Median	*p*-Value (Mann–Whitney)
SOD (µU/mg protein)	T0	455.0	1022.0	ns
	T1	331.5	366.4	ns
	T3	608.6	573.2	ns
	T6	478.1	764.9	ns
CAT (pmol/min/mg protein)	T0	1335.0	739.3	ns
	T1	664.8	483.0	ns
	T3	461.6	324.3	ns
	T6	327.2	375.6	ns
Px (mU/mg protein)	T0	4.836	4.917	ns
	T1	2.679	3.155	ns
	T3	3.502	3.885	ns
	T6	4.313	4.377	ns
GSH (ng/mg protein)	T0	6.514	6.263	ns
	T1	4.129	4.232	ns
	T3	4.705	4.638	ns
	T6	5.179	4.809	0.0893

SOD—superoxide dismutase; CAT—catalase; Px—peroxidase; GSH—reduced glutathione; ns—not significant (*p* > 0.05); PDT—photodynamic therapy; GKS—topical corticosteroids; T0—before treatment; T1—immediately after treatment; T3—3 months after treatment; T6—6 months after treatment.

**Table 7:** Mean, standard deviation, confidence interval, median, and minimum and maximum values of lesion sizes in group 1 (PDT) and group 2 (GKS) before treatment, immediately after treatment, and 6 months after treatment.

Time Point	Group	Mean (cm^2^)	SD	CI 95%	CI-95%	Median	Minimum	Maximum	*p* Value (Friedman Test)
T0	PDT	2.45	1.62	2.12	2.79	2.25	0.15	9.00	<0.0001
T1		1.07	1.03	0.86	1.28	1.00	0.00	4.00	
T6		0.69	1.05	0.47	0.90	0.00	0.00	4.00	
T0	GKS	2.31	1.92	1.85	2.78	2.03	0.35	12.00	<0.0001
T1		1.08	1.65	0.68	1.48	0.68	0.00	10.00	
T6		1.56	1.95	1.08	2.03	1.00	0.00	12.00	

**Table 8:** Median, minimum, and maximum VAS values as well as lower and upper quartiles in group 1 (PDT) and group 2 (GKS) before treatment, immediately after treatment, and 6 months after treatment.

Time Point	Group	Median	Minimum	Maximum	Lower Quartile	Upper Quartile
T0	PDT	3.00	2.00	10.00	3.00	5.00
T1		2.00	0.00	4.00	1.00	2.00
T6		1.00	0.00	3.00	0.00	1.00
T0	GKS	3.00	2.00	7.00	3.00	4.00
T1		1.00	0.00	5.00	0.00	2.00
T6		2.00	0.00	6.00	1.00	3.00

**Table 9:** The correlations between salivary antioxidant biomarkers and lesion size and pain intensity (VAS) in the PDT group at baseline (T0), immediately after therapy (T1), and at the 6-month follow-up (T6).

		Lesion Size		VAS	
Time Point	Biomarker	r	*p*	r	*p*
T0	SOD	0.093	0.522	−0.080	0.581
	Px	−0.222	0.121	0.107	0.461
	CAT	−0.157	0.277	−0.071	0.624
	GSH	−0.123	0.396	−0.010	0.944
T1	SOD	0.007	0.963	−0.007	0.962
	Px	−0.120	0.405	0.185	0.199
	CAT	−0.282	0.047	−0.311	0.028
	GSH	−0.086	0.551	−0.288	0.043
T6	SOD	0.032	0.825	−0.021	0.883
	Px	0.005	0.971	−0.083	0.567
	CAT	0.018	0.902	−0.085	0.557
	GSH	0.064	0.659	0.016	0.914

**Table 10:** The correlations between salivary antioxidant biomarkers and lesion size and pain intensity (VAS) in the GKS group at baseline (T0), immediately after therapy (T1), and at the 6-month follow-up (T6).

		Lesion Size		VAS	
Time Point	Biomarker	r	*p*	r	*p*
T0	SOD	−0.159	0.326	−0.0003	0.999
	Px	−0.035	0.828	0.341	0.031
	CAT	0.292	0.067	0.190	0.241
	GSH	0.018	0.914	0.338	0.033
T1	SOD	−0.183	0.258	−0.137	0.398
	Px	0.028	0.866	0.002	0.990
	CAT	0.304	0.057	−0.078	0.633
	GSH	0.169	0.297	−0.125	0.443
T6	SOD	0.172	0.289	0.215	0.182
	Px	0.039	0.810	0.115	0.481
	CAT	−0.060	0.715	−0.086	0.596
	GSH	0.122	0.453	0.086	0.599

## Data Availability

Data available on request due to restrictions privacy or ethical reasons.

## Supplementary Materials

The following supporting information can be downloaded at: https://www.mdpi.com/article/10.3390/ijms262211232/s1.
