# Supplementary material for: Time-Dependent Changes in Salivary Antioxidants After 5-ALA Photodynamic Therapy vs. Clobetasol in Oral Lichen Planus: A Randomized Clinical Trial
Source: Int J Mol Sci. 2025 Nov 20;26(22):11232. doi: 10.3390/ijms262211232 (PMC12653851; doi:10.3390/ijms262211232)
Supplement: Supplementary file 1 [file ijms-26-11232-s001.zip › ijms-3983494-supplementary.pdf]

Table S1. Dunn's post-hoc test. p-values for pairwise comparisons of SOD between time points (T0, T1, T3, T6) within therapies.

Comparison	PDT (p)	GKS (p)
T0 vs T1	>0.9999	0.0815
T0 vs T3	>0.9999	0.2783
T0 vs T6	>0.9999	>0.9999
T1 vs T3	0.4123	>0.9999
T1 vs T6	>0.9999	0.226
T3 vs T6	0.728	0.6547

Table S2. Dunn's post-hoc test. p-values for pairwise comparisons of CAT between time points (T0, T1, T3, T6) within therapies.

Comparison	PDT (p)	GKS (p)
T0 vs T1	0.009	0.0223
T0 vs T3	0.0052	0.0256
T0 vs T6	<0.0001	0.0127
T1 vs T3	>0.9999	>0.9999
T1 vs T6	0.8466	>0.9999
T3 vs T6	>0.9999	>0.9999

Table S3. Dunn's post-hoc test. p-values for pairwise comparisons of Px between time points (T0, T1, T3, T6) within therapies.

Comparison	PDT (p)	GKS (p)
T0 vs T1	<0.0001	<0.0001
T0 vs T3	0.2641	0.1461
T0 vs T6	>0.9999	>0.9999
T1 vs T3	0.0249	0.0721
T1 vs T6	<0.0001	0.0008
T3 vs T6	0.2189	>0.9999

Table S4. Dunn's post-hoc test. p-values for pairwise comparisons of GSH between time points (T0, T1, T3, T6) within therapies.

Comparison	PDT (p)	GKS (p)
T0 vs T1	<0.0001	0.002
T0 vs T3	0.199	0.3081
T0 vs T6	>0.9999	0.9951
T1 vs T3	0.0133	0.5993
T1 vs T6	<0.0001	0.1633
T3 vs T6	0.5302	>0.9999
